# TMS reveals inhibitory extrastriate cortico-cortical feedback modulation of V1 activity in humans

**DOI:** 10.1007/s00429-019-01964-z

**Published:** 2019-10-17

**Authors:** M. Maniglia, Y. Trotter, F. Aedo-Jury

**Affiliations:** 1 Centre de Recherche Cerveau et Cognition, Université de Toulouse, Toulouse, France; 2 Centre National de la Recherche Scientifique, Toulouse Cedex, France; 3 University of California at Riverside, Riverside, CA USA; 4 German Resilience Center (DRZ), Mainz, Germany

**Keywords:** Extrastriate feedback, Contextual modulation, Brain stimulation, TMS, Brain mechanisms

## Abstract

The interaction between the primary visual cortex (V1) and extrastriate visual areas provides the first building blocks in our perception of the world. V2, in particular, seems to play a crucial role in shaping contextual modulation information through feedback projections to V1. However, whether this feedback is inhibitory or excitatory is still unclear. In order to test the nature of V2 feedback to V1, we used neuronavigation-guided offline inhibitory transcranial magnetic stimulation (TMS) on V2 before testing participants on collinear facilitation, a contrast detection task with lateral masking. This contextual modulation task is thought to rely on horizontal connections in V1 and possibly extrastriate feedback. Results showed that when inhibitory TMS was delivered over V2, contrast thresholds decreased for targets presented in the contralateral hemifield, consistent with the retinotopic mapping of this area, while having no effect for targets presented in the ipsilateral hemifield or after control (CZ) stimulation. These results suggest that feedback from V2 to V1 during contextual modulation is mostly inhibitory, corroborating recent observations in monkey electrophysiology and extending this mechanism to human visual system. Moreover, we provide for the first time direct evidence of the involvement of extrastriate visual areas in collinear facilitation.

**Electronic supplementary material:**

The online version of this article (10.1007/s00429-019-01964-z) contains supplementary material, which is available to authorized users.

## Introduction

The interplay between striate and extrastriate visual areas shapes our perception of borders and contours (von der Heydt and Peterhans 1989), providing the basis for higher-level processes that rely on these early mechanisms. A key question in understanding the visual system is how units in early visual cortex, whose receptive fields are retinotopically organized, work in concert to give rise to a global and coherent perception of the world. A step toward understanding these dynamics is the discovery of contextual effects whereby neighboring units interact to signal the global properties of a stimulus (Nelson and Frost 1978; Gilbert and Wiesel 1990; Levitt and Lund 1997). Psychophysics studies stressed the role of collinearity, showing that these modulations are more evident when target and surround elements share the same local and global orientation, such as in contour integration (Field et al. 1993), crowding (Toet and Levi 1992), contrast facilitation (Polat and Sagi 1993), and orientation illusions (Kapadia et al. 2000). A number of these effects are thought to have its neural basis substrate in the striate cortex (V1), possibly involving modulatory effect from extrastriate feedback: Indeed, electrophysiology and brain stimulation studies offered evidence of such feedback on motion perception (Hupé et al. 1998), visual search (Juan and Walsh 2003), and surround suppression (Nassi et al. 2013), reporting the existence of feedforward–feedback recurring dynamics as well (Juan and Walsh 2003). A proposed role for this feedback mechanism is to help segregate low-contrast stimuli from the background by enhancing the inhibitory center–surround interaction. Moreover, feedback units with large receptive fields and faster conduction velocity would help connect distant regions of the surround that are beyond the spatial extent covered by intrinsic V1 connections (Angelucci and Bressloff 2006).

V2, the first extrastriate area, seems particularly involved in encoding orientation-related properties of the visual scene, i.e., occluded and illusory contours (von der Heydt and Peterhans 1989; Ramsden et al. 2001), motion contrast borders (Lu et al. 2010), and borders inferred from collinearity (Gilad et al. 2012). A recent monkey electrophysiology study (Nassi et al. 2013) showed that eliminating feedback from V2 induced response facilitation for stimuli extending beyond the receptive field center, consequently reducing surround suppression in V1. However, a previous monkey electrophysiological study reported suppression, rather than excitation, of V1 activity after extrastriate feedback removal (Hupé et al. 1998). Thus, the nature of V2 feedback to V1 during contextual modulation is still unclear in humans as in non-human primates. Collinear facilitation, the increase in contrast sensitivity for a target embedded in between two iso-oriented flankers (Polat and Sagi 1993), is another widely studied contextual modulation effect, observed both in fovea and near periphery (Maniglia et al. 2011, 2015a, b), thought to rely on similar early neural substrate as surround suppression, specifically the horizontal connections in V1 (Rockland and Lund 1982; Gilbert and Wiesel 1983, 1985; Polat and Sagi 1993). On the other hand, attention seems necessary for this effect to emerge (Freeman et al. 2001), and its range spans over several degrees of visual angle (Maniglia et al. 2015a, b) beyond the anatomical range of horizontal connections (Angelucci and Bressloff 2006), implying possible feedback from higher visual areas. Consequently, both connections in V1 and extrastriate feedback seem to be involved in collinear facilitation. However, up to now, no direct evidence of the latter has been reported.

Here, we measured collinear facilitation before and after neuronavigation-guided (Sack et al. 2009) offline repetitive transcranial magnetic stimulation (rTMS) on V2. rTMS can induce a temporary suppression of neural activity in the targeted area (Chen et al. 1997), allowing to draw casual relationships between stimulated region and performance. The use of the collinear facilitation paradigm (instead of, for example, surround suppression) served two purposes: testing whether or not extrastriate areas are involved in modulating this effect, as observed in monkey electrophysiology with a similar contextual modulation task and, if this is the case, disentangling the nature (excitatory vs inhibitory) of extrastriate cortico-cortical feedback to V1 during contextual modulation tasks in humans.

## Results

Since lateral interaction is a task known to be sensitive to practice, both in fovea and periphery (Polat and Sagi 1994; Maniglia et al. 2011), we analyzed the data as pre-/post-TMS stimulation contrast ratios.

**Fig. 1:**
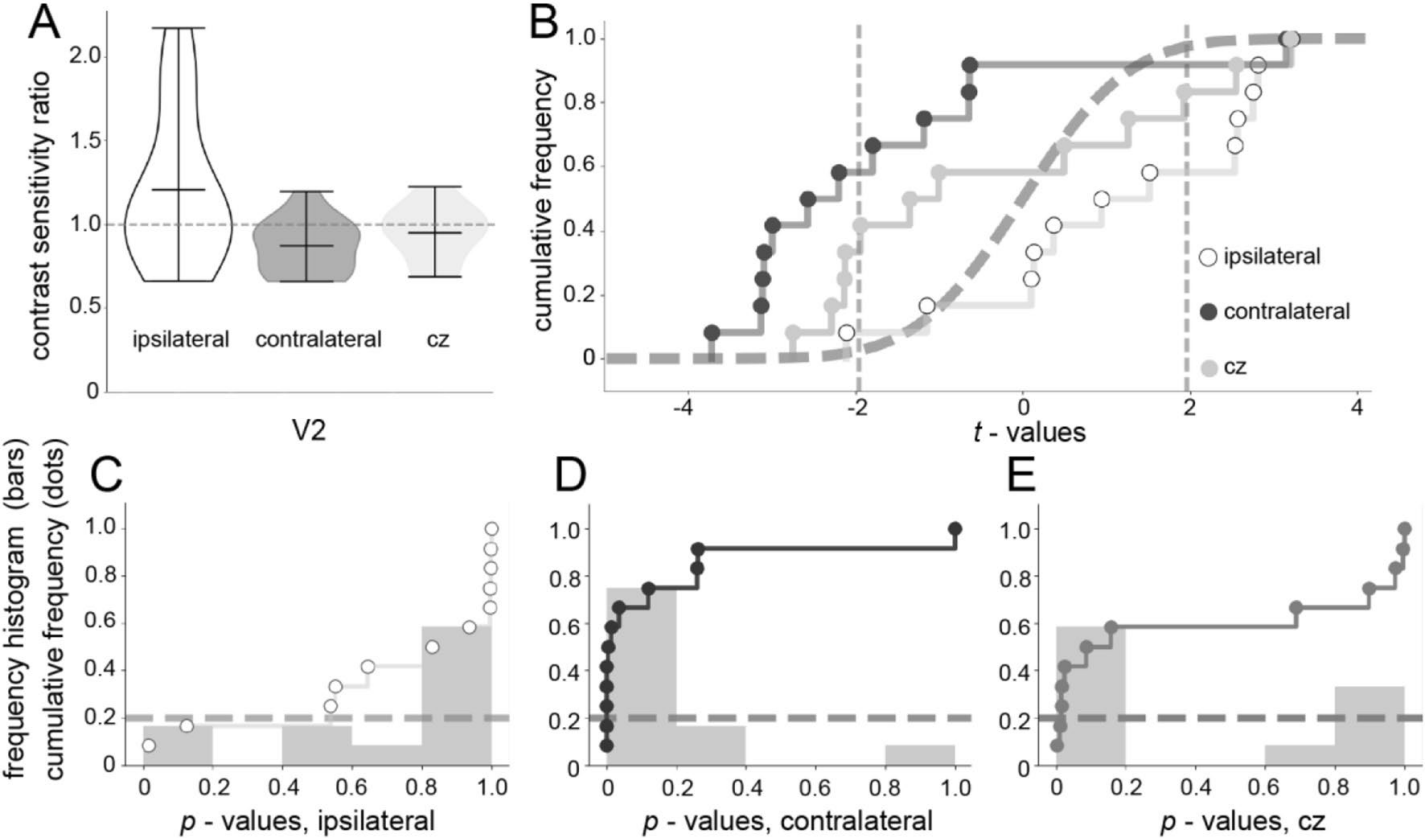
Behavioral results of the contrast sensitivity task. **a** Violin plots of the contrast sensitivity ratios between behavioral and stimulation session (*n* = 12 hemifield V2). Ratios were obtained as (pre-TMS thresholds/post-TMS threshold) for the contralateral (stimulated) and ipsilateral (non-stimulated) hemifield for the two V2 stimulation conditions, while for CZ, the ratio was obtained averaging contrast thresholds of the two hemifields. Horizontal central lines within each violin represent the mean of the CRS. Violin in white represents the CRS of the visual field ipsilateral respect to the TMS-stimulated one, while gray violin represents the contralateral. Dotted horizontal gray line indicates the 1:1 ratio. **b** Empirical cumulative distribution functions for the contrast sensitivity *z*-scores obtained across the 12 hemispheres for the comparisons between the visual field ipsilateral to the TMS stimulation (white), visual field contralateral to the TMS stimulation (dark gray), and control CZ (light gray). The dashed curve is the theoretical “chance” distribution (cumulative Gaussian with mean = 0 and sigma = 1). The two dotted vertical lines (*z*-scores of ± 1.96) define the regions of statistically significant differences (*p* < 0.05, two tailed) at the level of the individual participants. **c** Empirical cumulative distribution functions for the related contrast sensitivity test *p* values for the contralateral comparison. The histograms in the background have been obtained by grouping the *p* values in bins of 0.2 between 0 and 1. The gray and white areas indicate, respectively, the nonsignificant and significant *p* values (*p* < 0.05, two tailed) at the level of the individual participants. The dashed horizontal gray line represents the predicted uniform “chance” distribution of p values. **d** Same as (**c**) for the ipsilateral comparison. **e** Same as (**c**) for the control area CZ

rTMS significantly increased contrast thresholds in the contralateral hemifield (one-sample *t* test on pre-TMS/post-TMS ratios, *t*_11_ = 2.68, *p *= 0.021), while not affecting performance in the ipsilateral hemifield (*t*_11_ = 1.55, *p *= 0.14) or when delivered on the control region CZ (*t*_5_ = 0.67, *p *= 0.53) (Fig. 1a). We then tested contralateral vs ipsilateral hemifield ratio, observing a significant reduction in contrast thresholds for the former respect to the latter (paired *t* test, *t*_23_ = 2.29, *p* = 0.007). These results suggest that inhibiting V2 leads to an increase in contrast sensitivity in the contralateral (stimulated) visual field. Overall, there are both a significant post-TMS reduction for the contralateral hemifield and a difference between pre-/post-TMS ratios between the two hemifields. Therefore, the post-TMS increase in contrast sensitivity was significant for the retinotopically stimulated hemifield, but not for the ipsilateral, and the pre-/post-TMS ratio was significantly different between the two hemifields.

Figure 1a shows not only a change in the mean of the populations but also changes in the distribution of the dataset. In order to test whether the group effect obtained stands when analyzed on a subject by subject basis and to characterize the changes in the distribution of the dataset under different conditions, we ran a permutation test and then plotted the empirical cumulative distribution functions of the 12 hemispheres analyzed (Fig. 1b). Each hemisphere of the participants showed an independent behavior respect to its counterpart. (The contrast sensitivity for left and right eyes in the participant sample showed no dependence in both cases, when the eyes were contralateral (*Pearson correlation: r* = 0.42 *p* = 0.392*)* or ipsilateral to the later stimulation (*Pearson correlation: r* = − 0.11 *p* = 0.838*).*) Therefore, we considered both hemispheres as independent samples to the further analyses. (However, for the sake of completeness, we report the statistics conducted on subject—rather than on hemisphere based thresholds, that shows a significant reduction in contrast thresholds after offline TMS on the contralateral V2, one-sample *t* test vs 1, *t*_5_ = 3.59, *p* = 0.007, and no significant difference for the other comparisons: ipsilateral *t*_5_ = 0.55, *p* = 0.6, CZ *t*_5_ = 0.16, *p* = 0.88).

To further test the robustness of our group effect in single hemisphere, we ran a nonparametric permutation test. The two empirical distributions (ipsilateral and contralateral) were permuted between the control and the rTMS conditions in order to search for the effect of each condition in the distribution of the obtained values. The results show that both distributions differed markedly from each other and from the theoretical distribution, as confirmed by a nonparametric two-sample Kolmogorov–Smirnov test (KS-statistic = 0.75; *p *< 0.001). The ipsilateral visual field distribution (white) biases significantly from the theoretical distribution toward the right side, as attested by the results of a nonparametric Kolmogorov–Smirnov goodness-of-fit test (KS-statistic = 0.435; *p *= 0.01). On the other hand, the contralateral visual field distribution (gray) also differs markedly from that theoretical distribution but toward the right (white) side (KS-statistic = 0.653; *p* < 10^−5^). Together, these results indicate that both ipsilateral and contralateral visual fields changed their sensitivity to the collinear configuration after rTMS in opposite ways. These observations are reinforced by inspecting the distributions of *p* values associated with those *z*-scores (Fig. 1c–e). The *p* values distribution for the contralateral comparison (Fig. 1c) strongly differs from uniformity (KS-statistic = 0.538; *p* < 0.001). The inverse bias is observed in the ipsilateral condition (KS-statistic = 0.501; *p* = 0.002). The majority of the observations were significantly smaller in the post-TMS condition on the contralateral hemifield (9/12) and significantly larger in the ipsilateral hemifield (7/12). Altogether, these individual analyses give a clearer insight on the effect respect to those obtained at the group level with the *t* tests, corroborating an inverse effect on the contrast sensitivity when V2 is inhibited. Finally, we looked at the raw data scores for each session, separately for the two hemifields: Paired *t* test pre- vs post-TMS showed a significant effect of TMS in reducing contrast thresholds for the contralateral hemisphere (*t*_11_ = 3.67, *p* = 0.0037), while all the other comparisons were not significant.

## Discussion

The computation that takes place in V1 represents the first stage of visual analysis upon which more complex percepts are built. Recent evidence showed that even at this early stage, complex interactions take place, involving feedforward and feedback dynamics between V1 and extrastriate areas. However, it is still unclear how these mechanisms shape the initial perceptions. Contextual modulation, in which units with localized receptive fields interact to encode global properties of visual stimuli, constitutes the simplest form of these dynamics, therefore representing an ideal model. Moving from this, the aim of the present study was to solve two controversies: first, put to test the consistency of monkey electrophysiology with human brain cytoarchitecture; second, provide direct evidence of the involvement of extrastriate visual areas, specifically V2, in a contextual modulation effect, collinear facilitation, known to rely at least partially on the horizontal connection between hypercolumns in V1, and for which the role of extrastriate feedback has only been hypothesized so far.

Results showed that inhibitory rTMS over left or right V2 increased contrast sensitivity in the correspondent contralateral hemifield both respect to its pre-TMS level and to the ipsilateral hemifield pre-/post-TMS ratio. This suggests that V2 exerts inhibitory action during contextual modulation tasks that rely on interactions between receptive fields, consistent with recent animal electrophysiology evidence showing response facilitation and reduction in surround suppression in V1’s units when feedback from V2 was removed (Nassi et al. 2013).

Similarly, these results are consistent with a “predictive coding” type of theoretical framework (Mumford 1992; Rao and Ballard 1999), which suggests that higher-level units can recognize complex patterns thanks to their feedforward circuitry (Riesenhuber and Poggio 1999) and then selectively inhibit the local neural elements that represent partial portions of the pattern. The model then hypothesizes an iterative feedback–feedforward iteration, which gives rise to the final representation, discarded of the prediction. Indeed, our results show that the inhibition of extrastriate areas such as V2 prevents such a local inhibition from taking place, increasing contrast sensitivity as a result.

Given the difficulty in running neuroimaging-guided brain stimulation studies, the design of the present study has some limitations, mostly concerning stimulation propagation and lack of a control condition for the collinear configuration: Regarding the former, one could expect that the short eccentricity at which the experiment was conducted could add as a confound a potential stimulation of the striate cortex during the rTMS procedure. However, analysis of the TMS spread conducted with the software SimNIBS showed that the stimulation did not reach V1 (see Figure 1 supplementary material).

Additionally, the evidence that an increase in the contrast sensitivity in the contralateral hemifield was observed rules out this possibility: In fact, the inhibition of V1 is expected to produce a decrease rather than an increase in the contrast sensitivity (Amassian et al. 1989). Also, Merigan et al. (1993) demonstrated that a lesion in monkey V2 impairs complex orientation discrimination, but it does not affect visual acuity or contrast discrimination in relatively simple tasks, such as discriminating between horizontal and vertical gratings. In humans, transcranial magnetic stimulation (TMS) over V2 combined with TMS-induced electric field (E-field) modeling implies that V2 is necessary at least for discrimination of orientation of U-shaped hook (Thielscher et al. 2010). Moreover, we report for the first time direct evidence of the involvement of extrastriate visual areas in collinear facilitation. This is consistent with recent electrophysiological evidence and with a series of psychophysics studies who hinted at the role of extrastriate feedback in generating or modulating collinear facilitation (Huang and Hess 2008).

A possible, alternative explanation would be that inhibiting V2 had an indirect effect on V1 by disrupting a circuit that involves visual areas other than V2, however.

V2 is a strong candidate for being directly involved in generating collinear facilitation: (1) It has been shown to be involved in computing borders, edges, and in general contextual modulation phenomena (von der Heydt and Peterhans 1989; Ramsden et al. 2001; Lu et al. 2010; Gilad et al. 2012), and (2) a previous study in non-human primates suggested that in cases of perceptual effect involving contextual modulation, thus interaction and integration over multiple neurons’ receptive fields, V2 plays a crucial role (Nassi et al. 2013).

Concerning the lack of a control condition, it was beyond the scope of the present study to quantify threshold modulation due to collinear flankers respect to a baseline, as previous studies did (Polat and Sagi 1993; Maniglia et al. 2011). However, the parameters and eccentricity we used are known to elicit collinear facilitation; therefore, a change in contrast threshold following brain stimulation can most likely be attributed to an increase in the facilitatory effect of the flankers.

In conclusion, by using fMRI-guided neuronavigation to selectively inhibit V2 with offline rTMS, we showed for the first time the involvement of extrastriate areas in generating or modulating collinear facilitation. In particular, we observed that cortico-cortical feedback from V2 to V1 during contextual modulation tasks is mostly inhibitory.

## Materials and methods

### Apparatus

Stimuli were generated with MATLAB Psychtoolbox (Brainard 1997; Pelli 1997) and displayed on a 17″ Dell M770 CRT monitor, refresh rate 60 Hz. Screen resolution was 1024 × 768 pixels, each pixel subtending 1.9 arcmin. Mean luminance as measured with a Minolta CS110 (Konica Minolta, Canada) was 47.6 cd/m^2^. Bits# videocard (Cambridge Research Systems, Cambridge, UK) was used to increase the contrast range to 12 bits of luminance resolution. Images were linearized through a 12-bit gamma-corrected lookup table (LUT).

### Participants

Six participants (*n* = 12 since each hemifield was computed as an independent measure) with normal or corrected-to-normal vision took part in the experiment. The experimental protocol was approved by the relevant ethical committee at Centre National de la Recherche Scientifique with our institutional review board (CPP, Comité de Protection des Personnes, protocole 13018–14/04/2014) and was conducted in accordance with the Declaration of Helsinki (1964).

### Stimuli

**Fig. 2:**
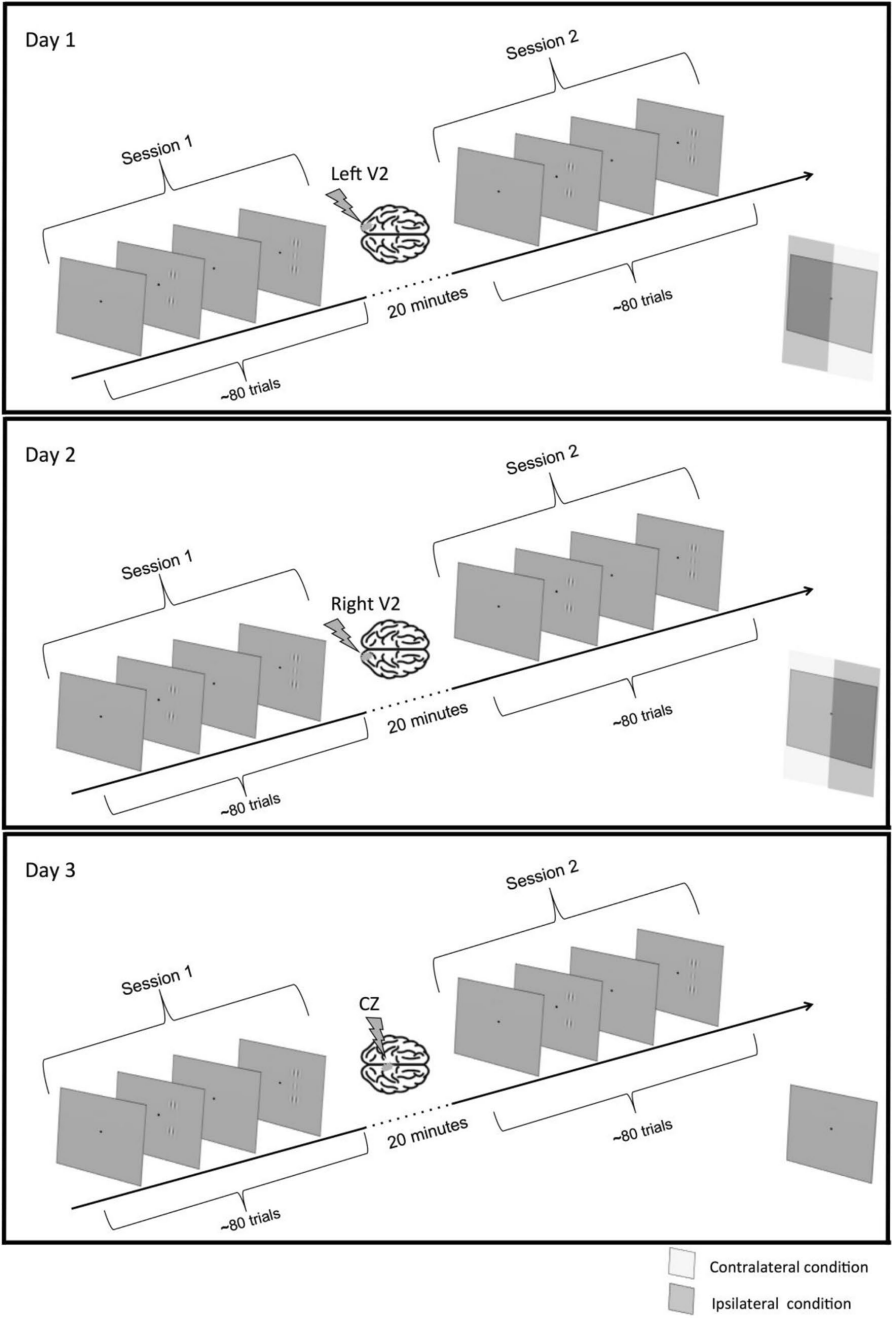
Experimental protocol. Participants underwent two sessions per day, for a total of six sessions (3 days). Each day started with a behavioral session of contrast detection with the lateral masking configuration presented 1.5° below the fixation point and 1.5° either left or right (counterbalanced and randomized within session). The two hemifields were defined as contralateral or ipsilateral according to the subsequent stimulation session, i.e., behavioral thresholds from left hemifield were considered “contralateral” in the session in which TMS was delivered over the right V2, and vice versa. For the control condition, we averaged contrast thresholds from both hemifields. Behavioral measurements were used to normalize contrast thresholds in the stimulation session and avoid confounds due to baseline inter-hemifield differences in contrast sensitivity. The session order was randomized (i.e., some participants started with TMS on left V2, others on right V2 or CZ)

Stimuli were Gabor patches, consisting of a cosinusoidal carrier enveloped by a stationary Gaussian. The configuration was composed by three Gabor aligned collinearly presented vertically. For all the conditions, *σ* = *λ* and spatial frequency is 3c/°. The location of the configuration target relative to the fixation point was 1.5° below and 1.5° either left or right (ipsilateral/contralateral hemifield). Gabor target was presented flanked above and below by two high-contrast (60%) Gabor patches arranged collinearly (Fig. 2). Target-to-flanker distance was 4*λ*.

### Method overview

Collinear facilitation is a perceptual task known to be sensitive to learning, both in fovea and near periphery (Polat and Sagi 1994; Maniglia et al. 2011, 2015a, b). To obtain more stable contrast thresholds and minimize the effect of learning that might occur in between days and affect our measurements, we had participants completing two daily sessions, one before and another one after rTMS, for a total of six sessions (two for each rTMS condition, see Fig. 2). Within each session, we randomly presented the stimulus configuration to the right or to the left of the fixation cross (1.5° below and 1.5° left/right), measuring separate thresholds for each side. We then considered these sides as contralateral or ipsilateral hemifield, according to where the TMS was delivered (i.e., right V2: left hemifield is contralateral; right hemifield is ipsilateral). We represented the data as ratios between these first and second daily sessions (TMS/behavioral), separately for each hemifield. Additionally, we selected a control region (CZ) to test for interactions between practice and nonspecific TMS effects, for which ratios were calculated by averaging between the two spatial locations (left and right).

#### Behavioral vs offline TMS: contralateral (experimental hemifield)

Our main hypothesis was that inhibiting V2 with low frequency, offline TMS would affect contrast detection of a flanked target in the corresponding contralateral retinotopic hemifield. Specifically, we expected contrast detection to improve after low-frequency offline TMS of V2, the rationale being that feedback from this region to V1 during a contextual modulation task appears to be mostly inhibitory, according to recent monkey electrophysiology evidence (Nassi et al. 2013). Therefore, reducing V2 activation, we expected a reduction in extrastriate inhibitory feedback and in turn an increase in contrast sensitivity.

#### Behavioral vs offline TMS: ipsilateral (control hemifield)

We used the ipsilateral hemifield as a control condition/region. Our hypothesis was that, being our stimulation site retinotopically correspondent to the contralateral hemifield, the respective ipsilateral hemifield would not be affected by the stimulation, thus acting as a within-session control location.

#### Behavioral vs offline TMS: CZ (control region)

To control for nonspecific effects of TMS on contrast sensitivity, we tested an additional control region, CZ, outside the occipital region.

### Procedure

Participants performed a contrast detection task for a central Gabor target flanked above and below by two high-contrast Gabor patches. Configuration was presented 1.5° below the fixation point, randomized of 1.5° left/right along the X-axis. Each session was composed by two interleaving, randomized staircases, separate for the left and the right hemifield. The procedure was a temporal two-alternative forced choice (2AFC), and participants had to report in which interval they saw the target by pressing a button on the keyboard (Fig. 2). Each interval lasted 80 ms, with a randomized inter-trial interval between 500 and 1000 ms. The target could be presented either in the left or right visual hemifield. The target was present only in one interval, while the flankers were presented in both. The target presentation hemifield was randomized on a trial basis. The target contrast varied according to a 3down/1up staircase starting from 1% contrast.

This value was then increased of 0.1 log units for each wrong response and decreased of the same amount after three consecutive correct responses. Each staircase (left and right) terminated after 80 trials or 15 reversals. The final threshold for each staircase, corresponding to 79% of correct responses, was obtained averaging the contrast threshold of the last 12 reversals. An acoustic feedback (50 ms tone of 500 Hz) was provided for wrong answers. Participants performed two daily sessions: one before TMS (*behavioral* session) and a second right after 20 min offline inhibitory 1 Hz rTMS (*stimulation* session). Each session lasted ~ 5 min. Each session was composed by two interleaved staircases in a single block. Participants performed six sessions overall distributed over 3 days (one *behavioral* and one *stimulation* per day), for a total of ten thresholds measurements (four *contralateral*, four *ipsilateral*, and two *control* thresholds). Participants sat in a dark room at a distance of 57 cm from the screen, and their head was stabilized using a chinrest. Viewing was binocular. They were instructed to fixate at the center of the screen where a fixation point was always present. In the analysis, due to our hypothesis, we refer to the hemifield as *contralateral* and *ipsilateral* with respect to the TMS-stimulated cortical region.

#### Permutation analysis

In order to overcome the limitations of the mean group analysis, the statistical significance of these contrast sensitivity values was evaluated for each participant through permutations tests. The 12 inversions obtained with the two versions of staircase procedure (pre- and post-TMS) under comparison were pooled before being randomly assigned to one or the other versions. We took the synthetic mean ratios among both groups of values. The same procedure was repeated 10.000 times. That way, we computed 10.000 synthetic ratios, from which we generated representative distributions of the ratios values that could have been obtained by chance. A *z*-score and a related *p* value were then obtained by dividing the observed ratio by the standard deviation of the Gaussian distribution generated by the permutation test (and always centered on ~ 1).

#### Impact of TMS at the population level

The distributions of ratios *z*-scores obtained for the test ipsilateral and contralateral were compared to each other with a two-sample Kolmogorov–Smirnov test. They were also, respectively, compared to the normal distribution expected in case of differences simply caused by random Gaussian noise (mean = 0 and standard deviation = 1) with one-sample Kolmogorov–Smirnov tests. The same procedure was applied with the related distributions of ratios *p* values, which were compared to each other and to the uniform distribution expected in case of Gaussian noise through one-sample and two-sample Kolmogorov–Smirnov tests. The analyses described above were implemented by using the Scipy 0.14 and Numpy 1.8.1 packages for Python (http://www.scipy.org) (Oliphant 2007a, b).

### TMS protocol and neuronavigation

In between the two daily sessions (*behavioral* and *stimulation*), participants underwent to 20 min of offline, low-frequency (1 Hz) TMS. Low-frequency TMS is thought to reduce cortical activity in the targeted area over a period of several minutes post-stimulation (Chen et al. 1997; Iyer et al. 2003; Battelli et al. 2009) by decreasing cortical excitability (Brignani et al. 2008).

In particular, in the visual cortex, low-frequency offline TMS has been shown to increase phosphene thresholds, a common evidence for reduced cortical excitability (Boroojerdi 2000). Recently, Khammash and colleagues (Khammash et al. 2019) showed that ppTMS affects similarly visual and motor cortex, in both cases inducing cortical inhibition.

**Fig. 3:**
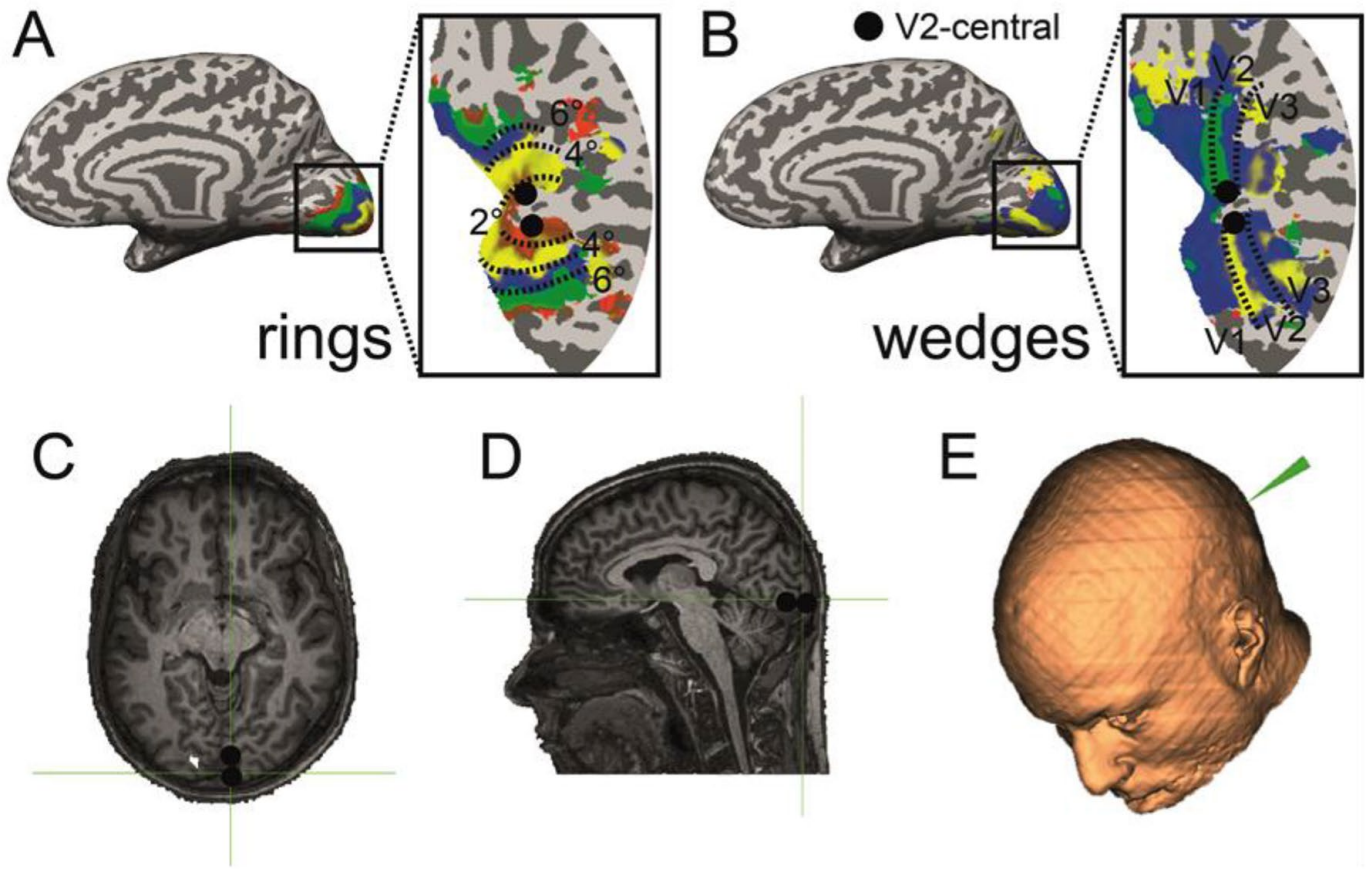
Retinotopic localization of the stimulated area. **a** Surface map of the right hemisphere of one participant showing the retinotopic maps obtained from the presentation of expanding/contracting rings. **b** Surface map of the same participants showing the retinotopic map obtained from the clockwise/anticlockwise wedges. The dorsal and ventral central spots of V2 identified with the maps are marked with a black circle. **c** Coronal view of the anatomical image of the participant with the 3D projection of the delimited V2 area. **d** Sagittal view of the same 3D projection. **e** 3D head reconstruction in the neuronavigation system and positioning of the TMS-stimulated spot corresponding to central V2. It can be observed that the cortical distance between left and right hemispheres is enough to discard any possible contamination due to the diffusion of the TMS pulse

Similarly to other papers (Battelli et al. 2009; Maniglia et al. 2012), we conservatively assumed the effect of offline TMS to be about 50% of the stimulation time. In total, 1200 pulses were delivered at an intensity of 50% maximum stimulator output (De Weerd et al. 2012). During the stimulation, the figure-of-eight-shaped coil (D 70 mm Alpha coil, Magstim) was held tight against the participant’s head by using a coil holder, and the participant’s head was propped up with a chinrest. Earplugs were used to attenuate the sound of the TMS pulse-induced noise. The coil plane was oriented tangentially to the scalp. The accuracy of TMS stimulation was ensured by an MRI-guided navigated brain stimulation (NBS) system (Brainsight™ 2 for TMS navigation) based on individual retinotopic mapping for each participant (Salminen-Vaparanta et al. 2012) (Fig. 3c–e). Additionally, the spread of the TMS was estimated with the software SimNIBS to ensure that the stimulation did not spread to visual areas other than V2.

This system uses ultrasound markers in a 3D space to track the position of the coil relative to the participant’s head. This is achieved by specifying fixed landmarks on the participant’s head, namely inion, nasion, and left and right ear. The neuronavigation system thus provides topographic information of the head-based transmitters relative to a participant-based coordinate frame. The same landmarks digitized on the participant’s head are specified on the head reconstruction of the anatomical MR data to achieve TMS–MRI coregistration. After coregistration, coil and head movements are registered online and are visualized in real time at correct positions relative to the anatomical reconstruction of the participant’s brain. The same system also permits the estimation of the distance between the center of the surface of the coil and the reconstruction of the cortical surface (coil–target distance) and the offset between the target location and the point of entry of the putative magnetic pulse “beam” on the cortical surface (beam–target distance). Through manual adjustment, we aimed to minimize these values during coil positioning and monitored these values for variations during the TMS pulse delivery. Thus, the use of fMRI-localizer-guided neuronavigation maximized the probability that the primary effect of the TMS pulses was in the target location in V2.

### Region localization

In order to localize the early visual areas (V1, V2, V3, and V3A), we performed standard retinotopic field mapping using clockwise/counterclockwise rotating wedges to define the cortical areas and expanding/contracting rings to define eccentricity (Fig. 3, Wandell et al. 2007). This methodology also called phase-encoded retinotopy is based on the traveling waves of BOLD signal activity created by the periodic stimulation of a particular portion of the visual cortex and varies smoothly across the cortical space generating contrast changes in the borders of the different areas (wedges) and at different eccentricities (rings), providing an accurate parcellation of the visual cortex. It has been widely used in visual sciences to characterize early visual cortical areas (for an extensive review see Wandell and Winawer 2011).

This mapping allowed us to define a portion of the visual field corresponding to each region within a single degree of eccentricity. For each of these four conditions, a run lasted 204 s and consisted of six full cycles of 32 s plus 12 initial s that permitted the establishment of the visual responses steady state. All the data were collected on a 3T scanner (Philips Achieva), using a standard 32 channels head coil. The functional data were acquired using (T2* weighted) echoplanar imaging (EPI). For the retinotopic mapping, we used: TR: 2 s; TE: 30 ms; field of view (FOV): 210 mm; voxel size 2 × 2 × 2 mm; no gap thickness; and SENSE factor: 2.5. A run comprised either 102 volumes (retinotopic mapping). A volume contained 33 slices that covered occipital and parietal cortices. We recorded eight runs for the retinotopic mapping experiment (two runs for each condition). The session also included the acquisition of a high-resolution anatomical image using a T1-weighted magnetization-prepared rapid gradient-echo (MPRAGE) sequence (160 slices; TR: 2300 ms; TE: 3.93 ms; FA: 12°; FOV: 256 mm; and voxel size 1 × 1 × 1 mm). This anatomical image was used as a reference to which the functional images from all the experiments were aligned.

#### Preprocessing

fMRI data were analyzed using the BrainVoyager QX software (v2.8, Brain Innovation) and MATLAB in-house built scripts. Preprocessing included slice scan time correction, 3D motion correction using trilinear/sinc interpolation, and high-pass filtering (0.01 Hz). For each individual participant, functional data were coregistered on the anatomy. Functional and anatomical data were brought into ACPC space using cubic spline interpolation. An in-house MATLAB script was used in order to obtain the averaged signal of the four runs of each condition in the wedges (two clockwise and two anticlockwise) and rings (two expanding and two contracting) and to create the file containing the values to be projected in the surface maps in BrainVoyager, where the central V2 spot was manually extracted based on the observed activations. Once marked the surface, this was projected back on the anatomical T1 using BrainVoyager (Fig. 3a, b).

## Electronic supplementary material

Below is the link to the electronic supplementary material.

Supplementary material 1 (DOCX 5764 kb)
