# Supplementary material for: TMS reveals inhibitory extrastriate cortico-cortical feedback modulation of V1 activity in humans
Source: Brain Struct Funct. 2019 Oct 17;224(9):3399–408. doi: 10.1007/s00429-019-01964-z (PMC6875154; doi:10.1007/s00429-019-01964-z)
Supplement: Supplementary file 1 — Supplementary material 1 (DOCX 5764 kb) [file 429_2019_1964_MOESM1_ESM.docx]

***Supplementary Material
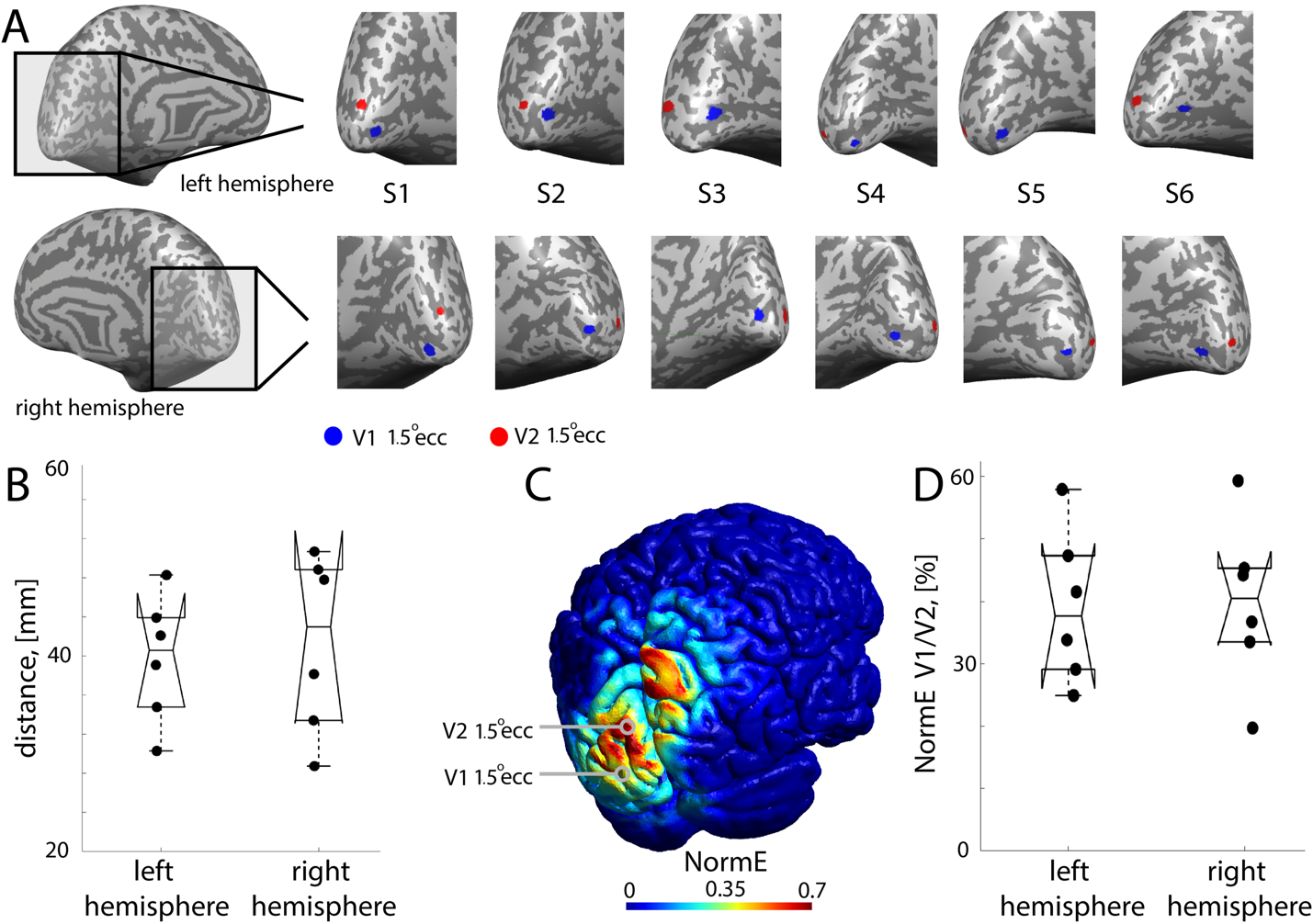
***

***Figure 1.* Distance between V1 and V2 at 1.5 degrees of eccentricity is enough to fade the TMS stimulation**. **A:** Surface maps of the 6 subjects used in the experiment marking the corresponding surface to 1.5 degrees of eccentricity in V1 (blue dot) and V2 (red dot) in the left (up) and right hemispheres (down) **B:** Shortest path calculation for each hemisphere of each subject. Distance were calculates using the shortest path calculation tool of BrainVoyager QX v2.8 (Brain Innovation, The Netherlands). The mean path calculated for the 6 subjects was: 49.87 mm ± 3.24 s.e.m. for the left and 40.63 ± 4.57 s.e.m. for the right hemisphere. **C:** To ensure that the effect of V2 stimulation on V1 was merely residual at the same eccentricity, we calculated and visualized the expected current density with the SimNibs software(1,2). In the figure we show the simulation of the rTMS stimulation on V2 in the left hemisphere for one exemplary subject with the norm of the electric field (NormE) that correspond to a smooth approximation of the electric field. **D:** Percentage of the norm of the electric field in V1 that arrives from V2 stimulation at 1.5 degrees of eccentricity in each hemisphere of each subjects. For the left hemisphere mean value is 39.1± 5.01 s.e.m. and for the right side 39.8± 5.42 s.e.m.

1. Thielscher, A., Antunes, A. and Saturnino, G.B. (2015), Field modeling for transcranial magnetic stimulation: a useful tool to understand the physiological effects of TMS? IEEE EMBS 2015, Milano, Italy.
2. Saturnino, G. B., Puonti, O., Nielsen, J. D., Antonenko, D., Madsen, K. H. H., and Thielscher, A. (2018), SimNIBS 2.1: A Comprehensive Pipeline for Individualized Electric Field Modelling for Transcranial Brain Stimulation. bioRxiv, 500314.


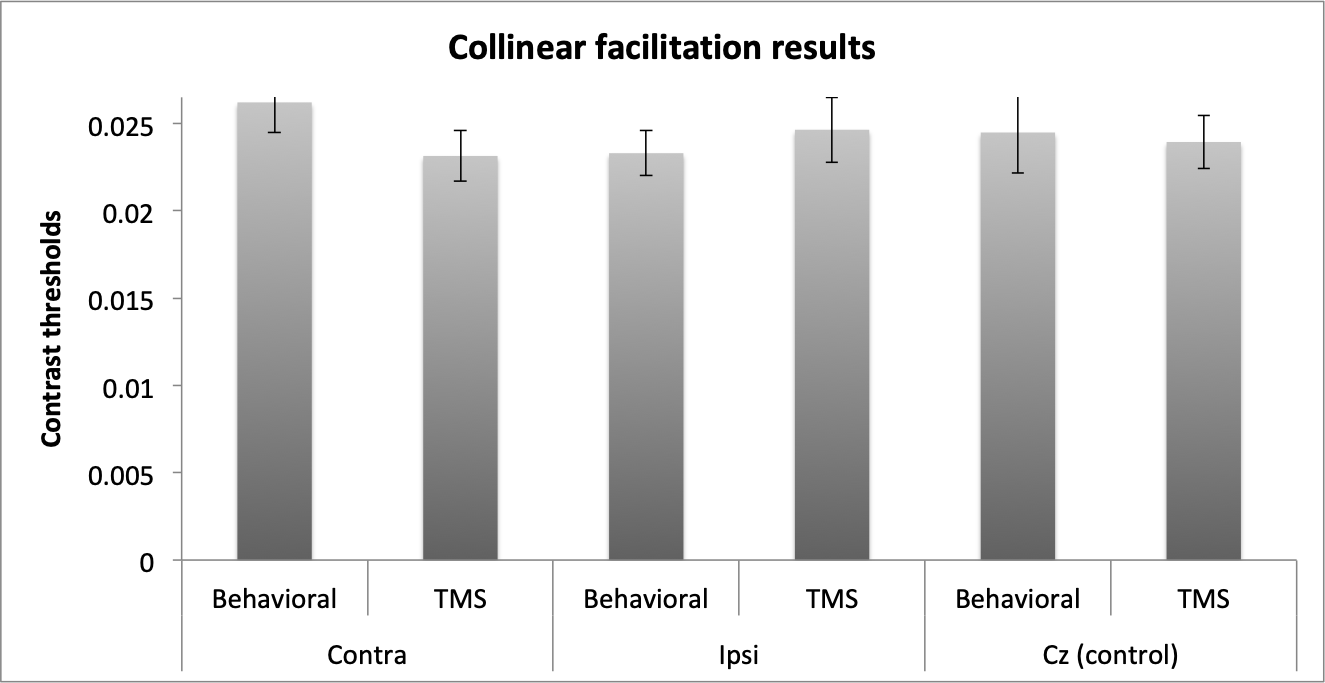


**Figure 2. Contrast thresholds for the 2 sessions (Behavioral and TMS) of the 3 conditions (Contralateral, Ipsilateral and CZ).** Error bars are +/- s.e.m.
